# Effects of Stem Cell Factor on Hypoxia-Inducible Factor 1 Alpha Accumulation in Human Acute Myeloid Leukaemia and LAD2 Mast Cells

**DOI:** 10.1371/journal.pone.0022502

**Published:** 2011-07-20

**Authors:** Bernhard F. Gibbs, Inna M. Yasinska, Abraham E. Oniku, Vadim V. Sumbayev

**Affiliations:** Medway School of Pharmacy, University of Kent, Kent, United Kingdom; University of Brescia, Italy

## Abstract

Stem cell factor (SCF) is a hematopoietic growth factor that exerts its activity by signalling through the tyrosine kinase receptor known as Kit or CD117. SCF-Kit signalling is crucial for the survival, proliferation and differentiation of hematopoietic cells of myeloid lineage. Furthermore, since myeloid leukaemia cells express the Kit receptor, SCF may play an important role in myeloid leukaemia progression too. However, the mechanisms of this pathophysiological effect remain unclear. Recent evidence shows that SCF triggers accumulation of the inducible alpha subunit of hypoxia-inducible factor 1 (HIF-1) in hematopoietic cells—a transcription complex that plays a pivotal role in cellular adaptation to low oxygen availability. However, it is unknown how SCF impacts on HIF-1α accumulation in human myeloid leukaemia and mast cells. Here we show that SCF induces HIF-1α accumulation in THP-1 human myeloid leukaemia cells but not in LAD2 mast cells. We demonstrated that LAD2 cells have a more robust glutathione (GSH)-dependent antioxidative system compared to THP-1 cells and are therefore protected against the actions of ROS generated in an SCF-dependent manner. BSO-induced GSH depletion led to a significant decrease in HIF-1α prolyl hydroxylase (PHD) activity in THP-1 cells and to near attenuation of it in LAD2 cells. In THP-1 cells, SCF-induced HIF-1α accumulation is controlled *via* ERK, PI3 kinase/PKC-δ/mTOR-dependent and to a certain extent by redox-dependent mechanisms. These results demonstrate for the first time an important cross-talk of signalling pathways associated with HIF-1 activation—an important stage of the myeloid leukaemia cell life cycle.

## Introduction

Stem cell factor (SCF) is a cytokine that plays a crucial role in hematopoiesis and melanogenesis [1]. It is recognised by the Kit receptor, also known as CD117 [1], [2], which is expressed in different types of non-differentiated hematopoietic cells including myeloblasts and pro-monoblasts [1]. Importantly, human myeloid leukaemia cells express the Kit receptor and their proliferation is upregulated by SCF [1]-[3], although mast cells are the only terminally differentiated hematopoietic cells that continue to express Kit [1], [3], [4].

In myeloid leukaemia cells SCF triggers accumulation of hypoxia-inducible factor 1 alpha (HIF-1α), an inducible subunit of the HIF-1 transcription complex [5]. HIF-1 is a heterodimeric protein containing a constitutive beta and an inducible alpha subunit. This complex plays a pivotal role in cellular adaptation to low oxygen availability as well as to the inflammatory stress associated with innate immune responses or allergic reactions [6]. The exact mechanisms leading to SCF-induced HIF-1α accumulation in myeloid leukaemia cells are unknown. However, it has been reported that mitogen-activated protein (MAP) and extracellular signal-regulating kinase (ERK) kinase (MEK) as well as phosphoinositide 3-kinase (PI3 kinase) but not p38 MAP kinase, play a role in SCF-induced HIF-1α accumulation in human myeloid leukaemia cells [5].

Since SCF acts *via* MEK, PI3 kinase and possibly protein kinase C (PKC) α/β isoforms [5], [7], one could hypothesise that SCF as such impacts on the activation of NADPH oxidase leading to the generation of reactive oxygen species (ROS) [6]. This assumption is supported by the fact that ROS seem to mediate some of the SCF-induced effects (e.g. glucose transport, synergy with granulocyte/macrophage colony-stimulating factor) [8], [9]. PKC α/β are known to activate NADPH oxidase by phosphorylating its component p47_phox_ [10], [11]. These events trigger HIF-1α accumulation, in most cases by affecting HIF-1α degrading prolyl-hydroxylases (PHDs) [6], [10], [12], [13]. However, mast cells, which are cultured in the presence of SCF and require this factor for support of their life cycle [14], do not suffer from permanent oxidative stress. One could suggest that the quantity of ROS produced by Kit-expressing cells in response to SCF is controlled by the glutathione-dependent anti-oxidative system. This includes γ-glu-cys-gly tripeptide known as glutathione (GSH) and several enzymes which produce GSH and which use it to scavenge ROS (glutathione peroxidase, GPx, which reduces peroxides using GSH as the electron/proton donor thus oxidising it to GSSG) and to reduce GSH, thus re-generating its antioxidative activity (glutathione reductase, GR) [15]. The robustness of the GSH antioxidative system in myeloid leukaemia cells and mast cells is, however, unknown and it is unclear whether this system and redox-dependent mechanisms in general play a role in SCF-induced HIF-1α accumulation.

Here, we studied the mechanisms of SCF-induced HIF-1α accumulation in THP-1 human myeloid cells. The key SCF-induced effects observed were compared to those in LAD2 human mast cells (which are normally cultured in the presence of SCF) upon normal presence or withdrawal of SCF. We report that SCF affects the GSH antioxidative system in THP-1 human myeloid leukaemia cells while inducing HIF-1α accumulation and a moderate increase in thiobarbiturate-reactive species (TBRS). SCF-induced HIF-1α accumulation was found to be a concentration-dependent process in THP-1 cells. Conversely, withdrawal of SCF did not significantly impact constitutive HIF-1α accumulation observed in LAD2 mast cells, although these cells expressed substantially higher amounts of GSH compared to those observed in THP-1 cells. No significant changes in TBRS were observed in LAD2 cells. The presence of SCF resulted in a very moderate decrease in HIF-1α PHD activity in both THP-1 and LAD2 cells. BSO-induced GSH depletion significantly decreased PHD activity in THP-1 cells and nearly attenuated it in LAD2 mast cells, suggesting an important protective function of GSH in both cell types, especially in mast cells. Further experiments have showed the role of PI3 kinase, ERK, PKC α, β and δ isoforms as well as mammalian target of rapamycin (mTOR) in HIF-1α stabilisation in THP-1 cells. On the other hand, NADPH oxidase-dependent ROS production was also found to contribute to the process to certain extent.

## Results

### SCF induces HIF-1α accumulation and affects the GSH antioxidative system in human myeloid leukaemia THP-1 cells but not in LAD2 mast cells

**Figure 1:**
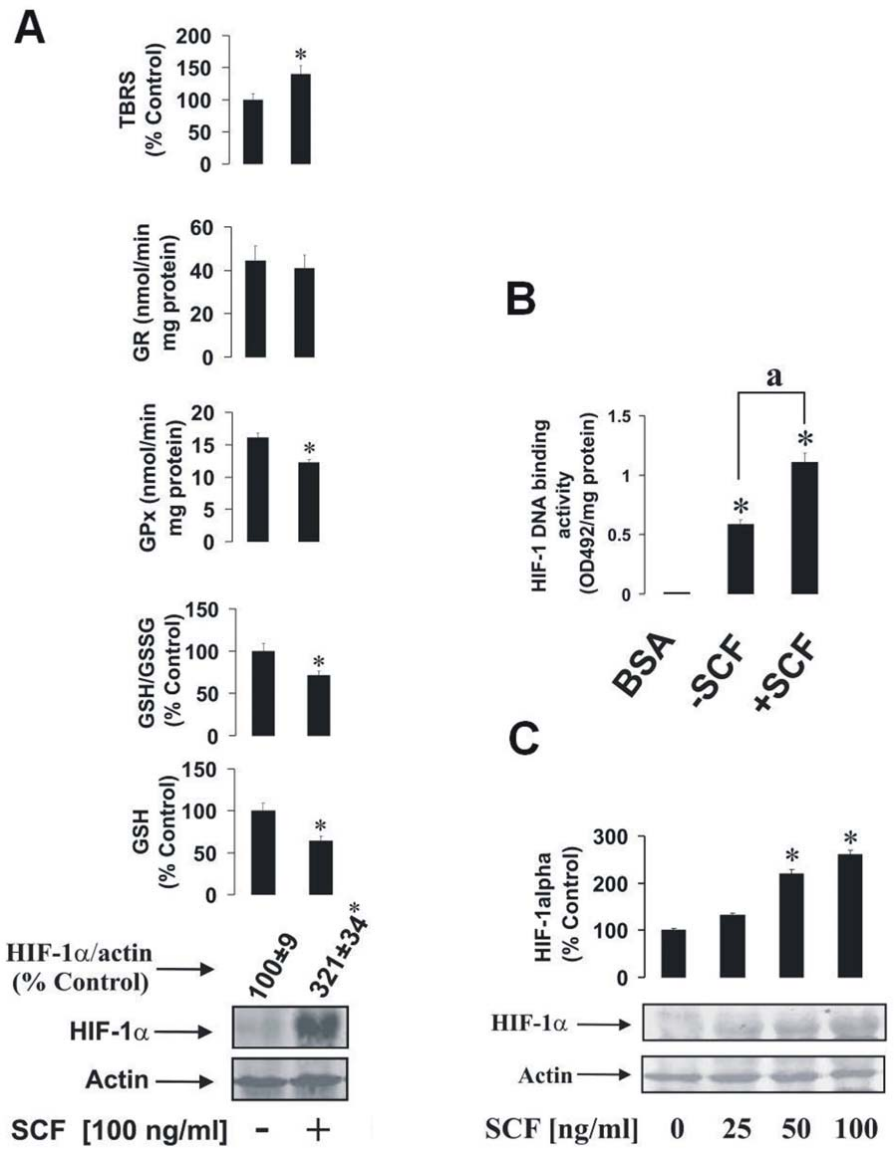
The impact of SCF on HIF-1α accumulation and the GSH-dependent antioxidative system in human myeloid leukaemia cells. (**A**) THP-1 human myeloid leukaemia cells were stimulated for 24 h with 100 ng/ml SCF. HIF-1α accumulation, GSH and GSSG levels, GPx/GR activities and the quantity of the TBRS were then measured as outlined in Materials and methods. (**B**) THP-1 human myeloid leukaemia cells were cultured in the presence or absence of 100 ng/ml SCF for 24 h. HIF-1 DNA-binding activity was analysed using 10 mg/ml BSA as a negative control. (**C**) THP-1 cells were exposed for 4 h to 25, 50 and 100 ng/ml SCF. HIF-1α accumulation was then measured as described in Materials and methods. Quantitative data are mean values ± S.D. of at least three individual experiments. *P<0.01 vs control. Western blot data are shown from one representative experiment out of three that gave similar results.

Firstly, we established whether SCF is able to induce HIF-1α accumulation and, at the same time, affects the GSH-dependent antioxidative system in THP-1 human myeloid leukaemia cells. We observed that SCF significantly upregulated HIF-1α accumulation in THP-1 cells following exposure to 100 ng/ml SCF for 24 h (Figure 1A). THP-1 cells also responded by a decrease in GSH production and a reduced GSH/GSSG ratio (Figure 1A). The presence of SCF reduced GPx activity, while no significant changes were observed in GR activity. TBRS levels were moderately increased in the presence of SCF (Figure 1A). Analysis of HIF-1 DNA-binding activity showed that the protein is transcriptionally active and significantly increased by 24 h incubation of THP-1 cells with SCF (Figure 1B). Furthermore, we observed that this SCF-mediated increase in HIF-1α accumulation was concentration dependent (Figure 1C).

**Figure 2:**
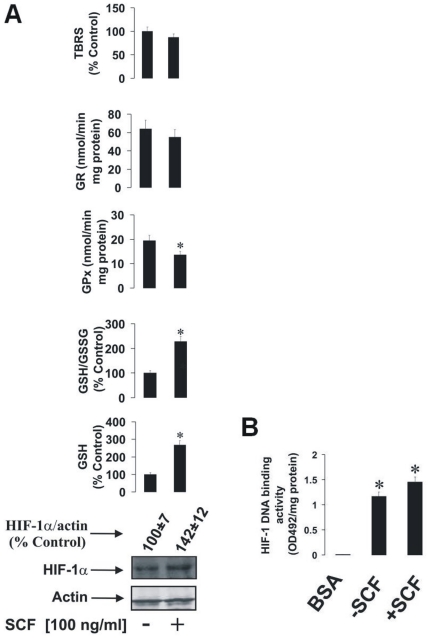
The impact of SCF withdrawal on HIF-1α accumulation and the GSH-dependent antioxidative system in LAD2 human mast cells. LAD2 cells (which were continuously cultured in the presence of 100 ng/ml SCF) were incubated in fresh media containing 100 ng/ml SCF for 24 h, while another fraction of these cells was subjected to SCF-free media for 24 h. (**A**) HIF-1α accumulation, GSH and GSSG levels, GPx/GR activities and the quantity of the TBRS were then measured as outlined in Materials and methods. HIF-1 DNA-binding activity was also analysed. (**B**) Quantitative data are mean values ± S.D. of at least three individual experiments. *P<0.01 vs control. Western blot data are shown from one representative experiment out of three that gave similar results.

Given the SCF-dependent effects on HIF-1α in THP-1 cells, which are a partially differentiated cell line, we wished to elucidate whether there is an overarching principle of SCF-mediated HIF-1α accumulation in terminally differentiated hematopoietic cells of myeloid lineage that still express functional Kit receptor. The only example of the latter is the mast cell. We therefore used LAD2 human mast cells, which display essential human mast cell properties [14]. These cells are normally cultured in the presence of SCF, thus excluding the possibility of direct comparisons to THP-1 cells. Despite this, however, we were able to determine whether withdrawal of SCF from the culture media could lead to downregulation of HIF-1α accumulation and associated effects in these cells. We therefore reserved one fraction of LAD2 cells that had been continuously cultured in the presence of 100 ng/ml SCF and incubated with fresh (SCF-containing) media for 24 h, while another fraction of these cells was cultured in SCF-free media for 24 h. In these settings, we were able to observe that SCF withdrawal for 24 h resulted in only a weak, and not significant, reduction in HIF-1α expression in LAD2 cells compared to those incubated with SCF (Figure 2A), although constitutive HIF-1α expressions in LAD2 cells cultured in the absence of SCF were higher than previously observed in THP-1 cells. In contrast to our previous data using THP-1 cells, LAD2 cells incubated in the presence of SCF showed increased GSH production compared to those cultured for 24 h without the cytokine (Figure 2A). The presence of SCF also led to reduced GPx activity, while no significant changes were observed in the activity of GR. TBRS level remained unchanged in LAD2 cells independently of SCF availability (Figure 2A). HIF-1 in LAD2 cells showed clear DNA-binding activity, which was not significantly changed upon SCF withdrawal (Figure 2B). Importantly, withdrawal of SCF neither affected LAD2 mast cell viability nor their capacity to generate vascular endothelial growth factor (VEGF; a HIF-1 downstream gene) as measured by ELISA (data not shown).

### GSH depletion leads to a significant decrease in HIF-1α PHD activity in both THP-1 and LAD2 cells

**Figure 3:**
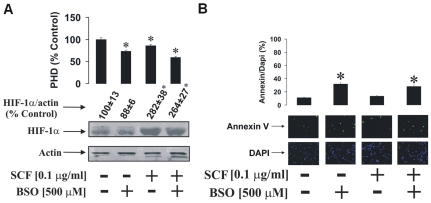
GSH depletion leads to a reduction of SCF-induced HIF-1α PHD activity in THP-1 cells. THP-1 human myeloid leukaemia cells were cultured (in fresh medium) for 24 h in the absence or presence of 100 ng/ml SCF ± 500 µM BSO. (**A**) HIF-1α accumulation/PHD activity and (**B**) annexin V/DAPI staining were performed as outlined in Materials and methods. Quantitative data are mean values ± S.D. of at least three individual experiments. *P<0.01 vs control. All Western blot and imaging data are from one representative experiment out of three that gave similar results.

Since ROS are likely to be produced in response to treatment with SCF [8], [9], we therefore investigated the role of the GSH-dependent antioxidative system in both THP-1 and LAD2 cells. THP-1 cells were stimulated for 24 h with 100 ng/ml SCF in the absence or presence of 500 µM buthionine sulphoximine (BSO) for 24 h. For control purposes, SCF-untreated cells also were exposed to 500 µM BSO for 24 h. We observed that, in THP-1 cells, the presence of BSO but in the absence of SCF led to decreased HIF-1α PHD activity (Figure 3A). In the presence of SCF, BSO also reduced PHD activity in THP-1 cells (Figure 3A). In parallel, HIF-1α accumulation in THP-1 cells was decreased by BSO in the absence of SCF. However, following 24 h exposure to SCF, HIF-1α accumulation was not affected by this inhibitor. The number of cells stainable with annexin V was significantly higher compared to the number of DAPI stainable cells in cells exposed to BSO independently of the presence or absence of SCF (Figure 3B).

**Figure 4:**
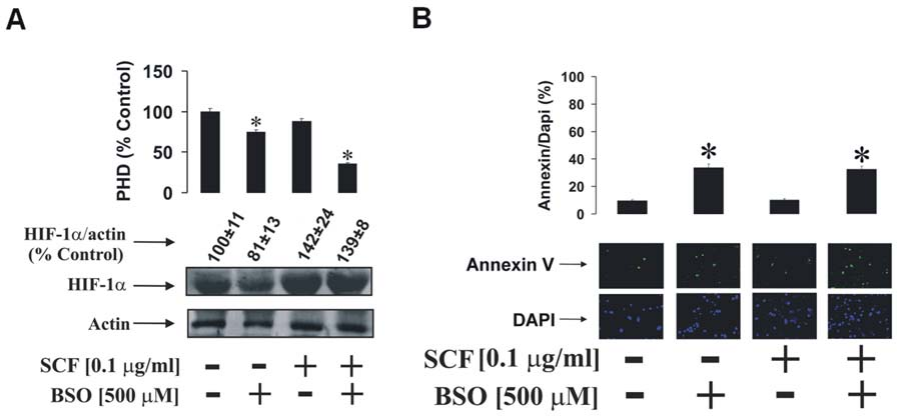
GSH depletion leads to a strong decrease in SCF-dependent HIF-1α PHD activity in LAD2 cells. LAD2 cells were incubated for 24 h in fresh media ±100 ng/ml SCF and ±500 µM BSO. (**A**) HIF-1α accumulation and PHD activity were assessed as well as (**B**) Annexin V and DAPI staining as outlined in Materials and methods. Western blot and imaging data are from one representative experiment out of three. Quantitative data are mean values ± S.D. of at least three individual experiments. *P<0.01 vs control.

We also compared the effects of BSO in LAD2 cells. For this purpose, we incubated one fraction of LAD2 cells in fresh media containing 100 ng/ml SCF and another fraction in fresh SCF-free media for 24 h. Both aliquots were incubated either with or without 500 µM BSO during this 24 h period. In the presence of SCF, BSO strikingly inhibited PHD activity (Figure 4A). HIF-1α accumulation in LAD2 cells was decreased in the presence of BSO in cells where SCF had been withdrawn. HIF-1α accumulation in cells exposed to SCF was not affected by BSO. As with THP-1 cells, the number of LAD2 cells stainable with annexin V was significantly higher compared to the number of DAPI stainable cells upon exposure to BSO independently of the presence/absence of SCF (Figure 4B).

This suggests that the cells begin to employ a PHD-dependent mechanism of HIF-1α accumulation in cases where GSH levels are affected. It may, however, also mean that GSH depletion chemically affects PHD activity by causing oxidation of PHD Fe^2+^, thus reducing its ability to hydroxylate HIF-1α [6], [13]. Decreased HIF-1α levels in cells exposed to BSO in the absence of SCF are probably controlled by apoptosis-dependent inactivation of some of the cells. Apoptotic changes might be caused by GSH depletion – this could be seen in the results of annexin V/DAPI staining presented in the Figures 3B and 4B.

### SCF-induced HIF-1α accumulation in THP-1 cells is regulated by several signalling pathways

**Figure 5:**
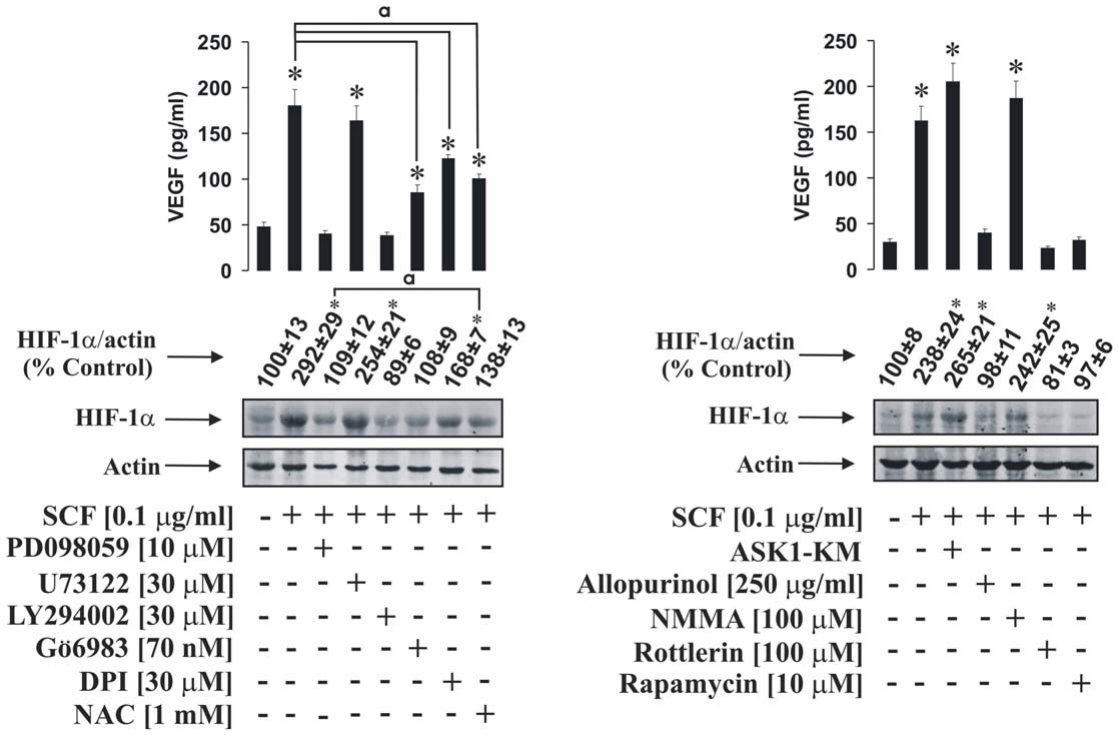
Signalling pathways involved in SCF-induced HIF-1α accumulation in THP-1 human myeloid leukaemia cells. THP-1 cells were pre-treated for one hour with the indicated concentrations of the outlined inhibitors. In one case cells were transfected with dominant-negative form of ASK1 (ASK1-KM) as indicated in Materials and methods. After that THP-1 cells were exposed for 4 h to 100 ng/ml SCF followed by Western blot analysis of HIF-1α accumulation and ELISA assay of the VEGF release. Quantitative data are mean values ± S.D. of at least three individual experiments. *P<0.01 vs control. **^a^** – differences are significant when comparing two indicated values (P<0.01). All Western blot data are from one experiment representative of three that gave similar results.

Since SCF was found to induce HIF-1α accumulation in THP-1 but not LAD2 cells, we investigated the biochemical mechanisms behind this process. We studied the contribution of several signalling pathways including ERK, phospholipase C 1 gamma (PLC-1γ the enzyme which is one of the upstream regulators of PKC α/β [8], [10]) and PI3 kinase. We also investigated the involvement of PKC α/β-induced NADPH oxidase activation leading to the generation of ROS. We first pre-treated THP-1 cells for 1 h with 10 µM PD098059 (ERK inhibitor) [12], 30 µM U73122 (PLC-1γ inhibitor), 30 µM LY294002 (PI3 kinase inhibitor), 70 nM Gö6983 (PKC α/β inhibitor), 30 µM DPI (NADPH oxidase inhibitor) and 1 mM NAC (ROS scavenging antioxidant, which also leads to an increase in intracellular GSH levels) [10], [12], [16], [17]. Cells were then exposed for 4 h to 100 ng/ml SCF. HIF-1α accumulation and production of VEGF (a HIF-1 downstream gene) were then analysed. We observed that PD098059 and LY294002 but not U73122 attenuated both SCF-induced HIF-1α accumulation and VEGF release (Figure 5). Gö6983, DPI and NAC downregulated both HIF-1α accumulation and VEGF production in THP-1 cells (Figure 5). One could therefore hypothesise that PI3 kinase, ERK and, to some extent, ROS, generated in a PKC α/β -NADPH oxidase-dependent manner contribute to SCF-induced HIF-1α accumulation in THP-1 cells, while PLC-1γ does not seem to participate. Given these results, we were interested to see whether apoptosis signal-regulating kinase 1 (ASK1), the ROS dependent pro-apoptotic upstream MAP kinase kinase kinase, could be involved in SCF-induced HIF-1α accumulation in THP-1 cells since Toll-like receptor 4-mediated processes are associated with cross-regulation of HIF-1α and ASK1 pathways [6], [10], [18], [19]. Four hours of exposure of THP-1 cells, transfected with the dominant-negative ASK1 isoform, to 100 ng/ml SCF led to further non-significant increases in HIF-1α accumulation supported by increased VEGF production (albeit not significant). We were also interested to see whether other reactive species-generating enzymes, which are normally upregulated in a cytokine–dependent manner, contribute to SCF-induced HIF-1α accumulation. These included xanthine oxidase (XOD, which converts hypoxanthine into xanthine and further into uric acid and produces superoxide radicals [20]–[22]), and nitric oxide synthase (NOS, generates nitric oxide [11]). We therefore pre-treated THP-1 cells with 250 µg/ml allopurinol (XOD inhibitor) [20] or 100 µM N-monomethyl-arginine (NOS inhibitor [10], [12]) and exposed them to 100 ng/ml SCF for 4 h. We found that allopurinol but not NMMA blocked both SCF-induced HIF-1α accumulation and VEGF production (Figure 5) suggesting that XOD but not NOS is involved in SCF-mediated processes.

The PI3 kinase pathway is known to upregulate HIF-1α accumulation in an mTOR-dependent manner [23], [24]. The serine/threonine protein kinase mTOR, which belongs to the PI3-kinase-related kinase protein family, contributes to regulation of protein synthesis and also transcription, thus impacting cell growth, proliferation, motility and survival [24], [25]. SCF is known to upregulate the delta isoform of PKC (PKC-δ) [25], which has also been shown to activate mTOR [26]. To address this question we pre-treated THP-1 cells for 1 h with 100 µM rottlerin (PKC-δ inhibitor [12], [25]) or 10 µM rapamycin (mTOR blocker) [23], [24] and then exposed them for 4 h to 100 ng/ml SCF. We found that both rottlerin and rapamycin completely attenuated SCF-induced HIF-1α accumulation and VEGF production in THP-1 cells (Figure 5) suggesting that these two enzymes are crucial for SCF-induced HIF-1α activation.

**Figure 6:**
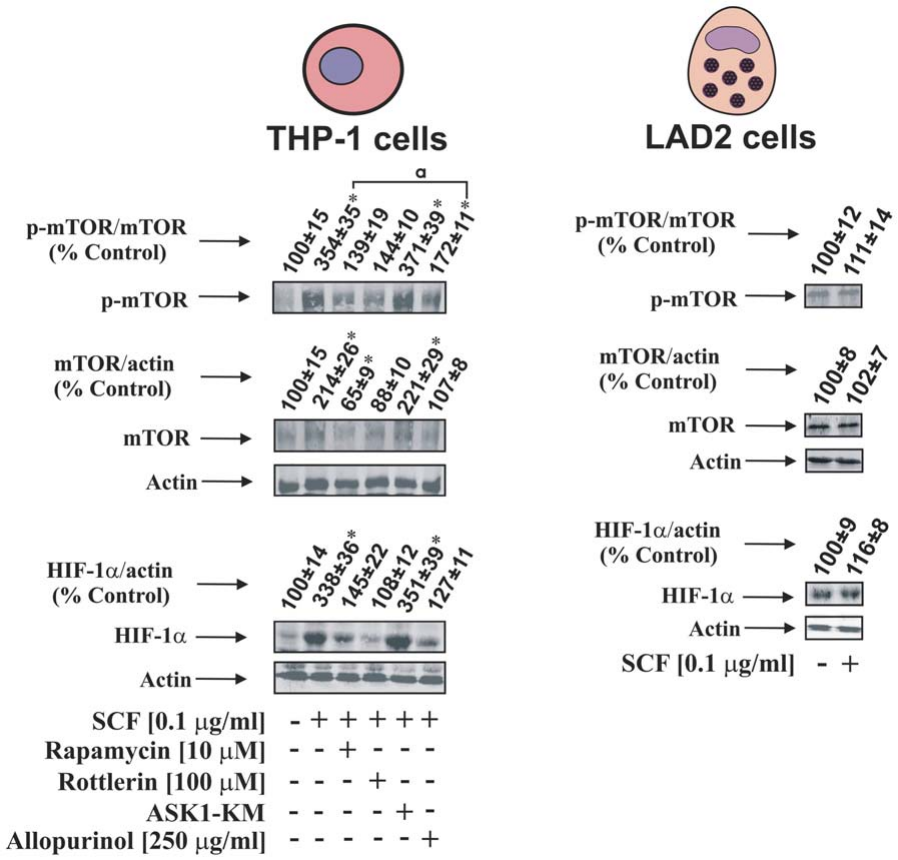
Several pathways impact SCF-dependent HIF-1α accumulation *via* mTOR in THP-1 human myeloid cells. THP-1 cells were pre-treated for one hour with the indicated concentrations of the outlined inhibitors. In one case cells were transfected with dominant-negative form of ASK1 (ASK1-KM) as indicated in Materials and methods. THP-1 cells were then exposed for 4 h to 100 ng/ml SCF followed by Western blot analysis of HIF-1α and mTOR accumulation as well as S2448 phosphorylation. LAD2 cells were cultured for 24 h in the absence or presence of 100 ng/ml SCF. All Western blot data are from one experiment representative of three that gave similar results. Quantitative data are mean values ± S.D. of at least three individual experiments. *P<0.01 vs control. **^a^** – differences are significant when comparing two indicated values (P<0.01).

Finally, we investigated the impact of ASK1 (as a pro-apoptotic negative regulator of protein synthesis), PKC-δ and XOD on mTOR accumulation and S2448 phosphorylation (XOD contributes to a crucial step of purine nucleotide catabolism, possibly influencing biochemical events associated with the whole transcription/translation system). In these experiments we pre-treated THP-1 cells with 10 µM rapamycin, 100 µM rottlerin or 250 µg/ml allopurinol for 1 h followed by 4 h of exposure to 100 ng/ml SCF. Some of the THP-1 cells were also transfected with the dominant-negative isoform of ASK1 and then exposed for 4 h to 100 ng/ml SCF. The accumulation of mTOR and HIF-1α protein were then analysed. We found that SCF clearly upregulated mTOR accumulation/S2448 phosphorylation in THP-1 cells. These processes were attenuated by rapamycin, rottlerin and allopurinol showing that both PKC-δ and XOD contribute to SCF-induced mTOR activation (Figure 6). Downregulation of ASK1 induced by its dominant-negative form led to a further non-significant increase in intracellular mTOR and its S2448 phosphorylation levels in THP-1 cells exposed to SCF. The effects of the above agents on the mTOR accumulation/S2448-phosphorylation correlated with those observed for HIF-1α (Figure 6). Interestingly, in LAD2 cells, mTOR was constitutively expressed and phosphorylated when they were cultured in the presence of 100 ng/ml SCF or kept for 24 h in the absence of this cytokine, which is consistent with absence in changes in HIF-1α accumulation (Figure 6).

## Discussion

SCF is recognised as a hematopoietic growth factor that exerts its activity by signalling through the type III receptor tyrosine kinase known as Kit receptor or CD117 [1]. SCF-Kit signalling is crucial for survival, proliferation and differentiation of hematopoietic cells of myeloid lineage [1]. Since myeloid leukaemia cells express the Kit receptor and are sensitive to SCF, this tyrosine kinase pathway has been suggested to contribute to leukaemia progression [1], [2], although, the molecular mechanisms of this pathophysiological SCF-Kit function remain unclear. SCF is also crucial for functioning of mast cells, which are the only terminally differentiated hematopoietic cells that express Kit receptors [1], [3], [4]. Recent evidence demonstrated that SCF induces accumulation of the inducible alpha subunit of HIF-1 transcription complex which limits its biological activity [5]. HIF-1 is crucial for cellular adaptation to low oxygen availability and angiogenesis, which form an important part of myeloid leukaemia progression [6], [27]. Since SCF acts *via* MEK, PI3 kinase and, possibly, PKC α/β isoforms, one could hypothesise that SCF as such impacts on the activation of NADPH oxidase leading to ROS generation. This event is known to reduce the activity of HIF-1α degrading PHDs [10], [13]. On the other hand, mast cells (for example LAD2) are cultured in the presence of SCF since they require this factor in principle [14]. But these cells are not known to suffer from permanent oxidative stress. One could hypothesise that the ability of SCF to induce the production of biochemically active ROS is controlled by a glutathione-dependent anti-oxidative system. It was, however, unknown how powerful this GSH antioxidative system in myeloid leukaemia and mast cells is. In addition, it was unclear whether this system, and redox-dependent mechanisms in general play a role in SCF-induced HIF-1α accumulation or the alternative mechanisms control the process.

To answer these questions we investigated whether SCF triggers HIF-1α accumulation in THP-1 human myeloid cells and in LAD2 mast cells. We also studied how SCF-Kit signalling impacts on the GSH-dependent antioxidative system and intracellular oxidative stress. Exposure of THP-1 cells to 100 ng/ml SCF significantly increased HIF-1α accumulation. This correlated with a decreased level of intracellular GSH and decreased GSH/GSSG ratio. GPx activity was also reduced with no significant changes in GR activity (see Figures 1A for details) indicating that the GSH-dependent system is affected in THP-1 cells under the influence of SCF. This conclusion was further supported by the observed SCF-dependent moderate increase in TBRS (Figure 1A). Removal of SCF from LAD2 mast cell cultures did not result in significant changes in HIF-1α accumulation compared to cells cultured in the normal manner with 100 ng/ml of the cytokine. However, this does not necessarily prove that SCF does not induce HIF-1α accumulation in LAD2 cells. Rather, the constitutive accumulation of HIF-1α could be close to the necessary functional threshold where any additional stimulation with SCF does not significantly increase its levels. Intracellular GSH levels and GSH/GSSG ratio were significantly higher when the SCF was present. The increase in GSH/GSSG ratio was, however, lower compared to the elevation of GSH, suggesting that GSSG levels also rose. GPx activity was reduced and GR activity was not affected. This suggests that LAD2 cells are by upregulating glutathione production in the presence of SCF (see Figure 2A). This effect in principle applies to both cell types (THP-1 and LAD2) since the amount of total glutathione (GSH+GSSG) was increased by SCF in both cases (321±38 % control in LAD2 cells and 182±17 % control in THP-1 cells). This might even permit the use of GSH as a direct ROS scavenger without employing the GSH-dependent enzymes in these cells. In LAD2 cells exposed to SCF, TBRS levels were not changed compared to cells cultured in the absence of SCF. In both cell lines HIF-1 was transcriptionally active (Figures 1B and 2B). We also found that in THP-1 cells SCF-induced HIF-1α accumulation is a concentration-dependent process (see Figure 1C). It is important to mention that a high level of basic HIF-1α accumulation in LAD2 mast cells was observed independently of the presence of SCF. This is possibly a result of a high level of glycolysis in these cells [28], [29]. HIF-1 is known to control glycolysis on the transcriptional level [27], with ATP produced *via* glycolysis and thereby playing a crucial role in mast cell function [28].

We therefore investigated the role of GSH in regulation of HIF-1α accumulation and PHD activity in both THP-1 and LAD2 cells. Cells were exposed to 500 µM BSO in the absence or presence of 100 ng/ml SCF. In THP-1 cells we observed a reduction of PHD activity in the BSO treated cells in the absence of SCF. HIF-1α accumulation was non-significantly decreased, which is probably a result of the fact that some of the BSO treated cells underwent apoptotic changes (Figure 3A and B). In the presence of SCF, BSO led to a strong reduction in PHD activity. However, SCF-induced HIF-1α accumulation was not affected, while the numbers of apoptotic cells were increased compared to experiments when the cells were exposed to SCF in the absence of BSO (see Figure 3A and B). Similar effects were observed in LAD2 cells but differed in that SCF did not lead to a significant increase in HIF-1α accumulation. Furthermore, BSO induced apoptosis of a higher number of LAD2 cells and in the presence of both BSO and SCF, PHD activity was substantially attenuated (Figure 4A and B). One could therefore assume that the GSH-dependent system is more powerful in LAD2 mast cells compared to that of THP-1 myeloid leukaemia cells. BSO-induced reduction in PHD activity, especially in the presence of SCF, might be a compensatory mechanism which supports the generation of the required level of HIF-1α in these cells. Alternatively, oxidation of the PHD catalytic site may also lead to a steady level of HIF-1α. However, it is important to mention that the decrease in PHD activity induced by SCF was minor especially in LAD2 cells (see Figures 3A and 4 A). This suggests a minor contribution of PHD to SCF-induced HIF-1α accumulation.

Mechanistic experiments using pharmacological inhibitors of a number of signalling enzymes suggested that ERK is involved in SCF-induced HIF-1α accumulation/activation in THP-1 cells. While PLC-1γ did not seem to affect the process, the PKC α/β-NADPH oxidase-ROS branch was found to contribute to the SCF-dependent HIF-1α accumulation and activation (given the respective values of released VEGF). However, this contribution was moderate compared to the role of other pathways. A crucial role for PI3 kinase, PKC-δ and mTOR was observed. PI3 kinase and also PKC-δ are known to be activated in SCF-dependent manner [1], [5], [25], [26]. Both pathways appear to activate mTOR and blocking mTOR with rapamycin or inhibition of any of these kinases – PI3 kinase or PKC-δ strongly attenuated SCF-induced HIF-1α accumulation in THP-1 cells (Figure 5).

Interestingly, the XOD inhibitor allopurinol, but not NMMA – a NOS inhibitor, also decreased SCF-induced HIF-1α accumulation. Attenuation of ASK1 activity by transfection of THP-1 cells with dominant-negative ASK1 isoform led to a further increase in SCF-induced HIF-1α accumulation (Figure 5).

mTOR was found crucial for SCF-induced HIF-1α accumulation in THP-1 cells, which actually confirmed previous observations suggesting translational nature of SCF-induced HIF-1α activation [5]. We therefore investigated whether the most effective inhibitors are able to affect mTOR as well as its S2448 phosphorylation and found that levels of accumulated HIF-1α and mTOR/phospho-S2448-mTOR correlated (see Figure 6). Rapamycin and rottlerin (PKC-δ inhibitor) led to attenuation of SCF-induced mTOR/phospho-S2448-mTOR levels; the same effect was also observed for the XOD inhibitor (Figure 6). Since XOD contributes to a crucial step of purine nucleotide catabolism controlling their utilisation and re-use, it might influence biochemical events associated with the whole transcription/translation system. Downregulation of ASK1 activity achieved by transfection of the cells with its dominant-negative isoform led to a further non-significant increase in mTOR/phospho-S2448-mTOR levels, which correlated with the effect observed on HIF-1α accumulation (see Figure 6). Such an effect could be possibly explained by the pro-apoptotic nature of the redox-dependent ASK1 activity in the cells [30]. The lack of significant differences in this case could also be a result of the respective protein levels reaching the thresholds required for their specific functions. Interestingly, in LAD2 cells, mTOR was constitutively expressed and phosphorylated when the cells were cultured either in the presence of 100 ng/ml SCF or kept for 24 h in the absence of this cytokine. These observations are consistent with a previously published observation [31] as well as the absence of changes in HIF-1α accumulation seen in the present study (see Figure 6).

**Figure 7:**
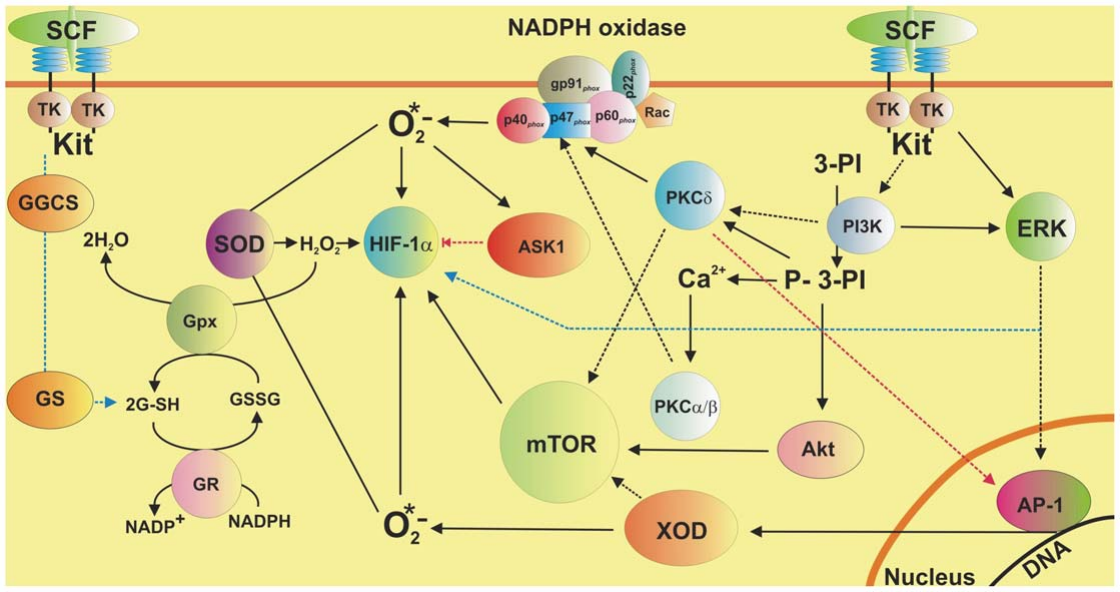
Possible mechanisms of SCF-dependent HIF-1α accumulation in human myeloid leukaemia cells. Implication of a GSH-dependent antioxidative system. The scheme demonstrates the involvement of ERK, PI3 kinase/PKC-δ-mTOR, PKC α/β-NADPH oxidase-ROS and XOD pathways in SCF-induced Kit receptor-dependent HIF-1α accumulation in THP-1 human myeloid leukaemia cells. The implication of GSH-dependent antioxidative system and ASK1 in the process is outlined.

We conclude that SCF leads to a significant accumulation/activation of HIF-1α in human myeloid leukaemia THP-1 cells but not in LAD2 human mast cells. LAD2 cells employ a more powerful GSH-dependent antioxidative system compared to THP-1 myeloid leukaemia cells. LAD2 mast cells are therefore completely protected against action of ROS generated in SCF-mediated manner. In both cases GSH plays an ROS scavenging role. BSO-induced GSH depletion leads to a significant decrease in PHD activity in THP-1 cells and to near attenuation of it in LAD2 cells. In THP-1 cells SCF-induced HIF-1α accumulation is controlled *via* ERK, PI3 kinase/PKC-δ/mTOR-dependent and to certain extent by a redox-dependent mechanisms. Additionally, XOD contributes to SCF-induced mTOR accumulation, while it is also known to produce superoxide anion-radicals. These mechanisms are summarised in the scheme presented in the Figure 7.

In summary, our results demonstrate the mechanisms involved in SCF-induced HIF-1 activation in human myeloid leukaemia cells. HIF-1 is crucial for cellular adaptation to low oxygen availability [27] and hypoxia is a physiological environment for cancer progression including myeloid leukaemia [6], [27]. Given this, our results demonstrate for the first time an important cross-talk of signalling pathways associated with this crucial stage in the myeloid leukaemia cell life cycle.

## Materials and Methods

### Materials

RPMI-1640 medium, foetal calf serum and supplements, DOTAP transfection reagent, GSH, GSSG and BSO were purchased from Sigma (Suffolk, UK). Maxisorp™ microtitre plates were obtained from Nunc (Roskilde, Denmark). ELISA-based assay kit for detection of VEGF was bought from R&D Systems (Abingdon, UK). Mouse monoclonal antibodies to HIF-1α, mTOR and β-actin as well as rabbit polyclonal antibody against phospho-S2448 mTOR were from Abcam (Cambridge, UK). Goat anti-mouse and goat anti-rabbit fluorescence dye-labelled antibodies were obtained from Li-Cor (Lincoln, Nebraska USA). Stem-Pro-34 serum-free media and SCF were obtained from Invitrogen (Paisley, UK). Biotinylated peptide (biotin-DLDLEMLAPYIPMDDDFQL – the peptide corresponds to the region of HIF-1α protein which is between 556^th^ and 574^th^ amino acid residues) – the substrate of HIF-1α PHD was obtained from Biopeptide Co. (San Diego, CA, USA). All other chemicals were of the highest grade of purity and commercially available.

### LAD2 mast cells

LAD2 mast cells were kindly provided by A. Kirshenbaum and D. Metcalfe (NIH, USA). Cells were cultured in the Stem-Pro-34 serum-free media with supplement containing 2 mM L-glutamine, penicillin (100 units/ml), streptomycin (100 µg/ml) and recombinant human SCF (100 ng/ml) [14].

### THP-1 human myeloid cells

THP-1 human leukaemia monocytic macrophages were purchased from the European collection of Cell Cultures (Salisbury, UK). Cells were grown in RPMI 1640 media supplemented with 10% foetal calf serum, penicillin (50 IU/ml) and streptomycin sulphate (50 µg/ml).

### Plasmids

Plasmid encoding hemagglutinin (HA)-tagged human ASK1 with kinase-dead domain (dominant-negative form, ASK1-KM) was a kind gift of Professor H. Ichijo (University of Tokyo, Tokyo, Japan). Plasmid was amplified in E. Coli XL10 Gold® (Stratagene Europe, Amsterdam, The Netherlands) and purified using GenElute™ plasmid purification kit according to the manufacturer's protocol. Purified plasmids were transfected into THP-1 cells using DOTAP transfection reagent according to the manufacturer's protocol. Successful transfection was monitored by Western blot [10], [30] (3±0.6 fold of increase in ASK1 was observed in the transfected cells) and detection of its kinase activity [10], [30], which was attenuated in all experiments – as shown in our previous reports [10], [30].

### Western blot analysis

HIF-1α mTOR and phospho-S2448 mTOR were determined by Western blot analysis, as previously described [10], [12]. Briefly, cells were incubated for the times indicated, washed twice with ice-cold phosphate-buffered saline (PBS) and lysed in 200 µl of lysis buffer (50 mM Tris–HCl, 5 mM EDTA, 150 mM NaCl, 0.5% Nonidet-40, 1 mM PMSF, pH 8.0). After centrifugation (13000 rpm, 15 min) the protein content in the supernatants was analysed. Finally, 40 µg of protein was added to the same volume of 2× sample buffer (125 mM Tris–HCl, 2% sodium dodecyl sulfate (SDS), 10% glycerine, 1 mM dithiothreitol (DTT), 0.002% bromphenol blue, pH 6.9) and boiled for 5 min. Proteins were resolved on 7.5% SDS–polyacrylamide gels and blotted to nitrocellulose membranes. Molecular weights were calibrated in proportion to the running distance of rainbow markers. HIF-1 or mTOR antibodies were added and incubated for 60 min at room temperature. Afterwards, nitrocellulose membranes were washed five times for 15 min and subjected to incubation with Li-Cor (Lincoln, Nebraska USA) goat secondary antibodies conjugated with fluorescent dyes for 60 min according to the manufacturer's protocol. β-actin staining was used to confirm equal protein loading as described previously. The Western blot data for HIF-1α protein were subjected to a quantitative analysis using Odyssey software and values were normalised against respective actin bands.

### Determination of HIF-1 DNA-binding activity

HIF-1 DNA-binding activity was measured by the method similar to the one described recently [10], [12]. A 96-well maxisorp™ microtitre plate was coated with streptavidin and blocked with BSA. 2 pmol/well biotinylated 2HRE-containing oligonucleotide were immobilised by 1 h incubation at room temperature. The plate was then washed 5 times with TBST buffer (10 mM Tris-HCl, pH 8.0, 150 mM NaCl, 0.05% Tween-20) followed by 1 h incubation with 20 µl/well of cell lysate at room temperature. The plate was again washed 5 times with TBST buffer and mouse anti-HIF-1α antibody (1∶1000 in TBS plus 2% BSA) was added. After 1 h of incubation at room temperature the plate was washed 5 times with TBST buffer and incubated with 1∶1000 HRP-labelled rabbit anti-mouse IgG in TBST buffer and, after extensive washing with TBST, the bound secondary antibody was detected by the peroxidase reaction (*ortho*-phenylenediamine/H_2_O_2_, Kem-En-Tek Diagnostics, Copenhagen, Denmark). Reactions were quenched after 10 min with an equal volume of 1 M H_2_SO_4_ and the colour development was measured in a microplate reader (absorbance at 492 nm). DNA-binding activity of HIF-1 was calculated as OD492 per mg protein present in the cell lysates compared to 10 mg/ml BSA (negative control).

### Measurement of VEGF production

Production of VEGF by the THP-1 cells was analysed by ELISA according to the manufacturer's protocols.

### Analysis of HIF-1α PHD activity

To detect HIF-1α PHD activity we employed a peptide-based assay as described before [13], [32]. For the assay we used HIF-1α-free cell lysates to avoid the impact of hydroxylation of the intracellular HIF-1α. Lysates of non-treated and treated THP-1 cells were incubated for one hour in the 96-well maxisorp™ microtitre plates, which were coated with HIF-1α capture antibody and blocked with BSA as described before [13]. Upon completion of the incubation lysates were used for the PHD assay.

#### Peptide-based assay

A 96-well maxisorp™ microtitre plate was coated with streptavidin and blocked with BSA as described before [13]. 100 ng/well of biotinylated peptide DLDLEMLAPYIPMDDDFQL was then immobilised as described. The plate was washed 3 times with TBST buffer (10 mM Tris-HCl, pH 8.0, 150 mM NaCl, 0.05% Tween-20). Then HIF-1α-free cell lysates (where the PHD activity was tested) were added to the wells together with the 20 mM Tris PHD assay buffer (pH 7.5) containing also 5 mM KCl, 1.5 mM MgCL_2_, 100 µM 2-OG, 10 mM FeSO_4_ and 2 mM ascorbate in a final volume 120 ml. The plates were incubated for 60 min at room temperature and the reaction was stopped by washing the plate 3 times with the TBST buffer. We then measured the binding of pVHL to the peptide [13], [32]. PHD activity in the non-treated THP-1 cells normalised against the amount of the intracellular protein detected by Bradford assay was counted as 100 %. Respectively, the activity in other samples was expressed as % control.

### Glutathione determination

Cells were lysed by the addition of 150 µl 1% sulfosalicyl acid and incubation for at least 10 min at 4°C. After centrifugation (10,000×g, 5 min), 130 µl of the supernatant was further processed for GSSG quantification. GSH content was directly measured. For GSSG analysis, reduced glutathione was derivatised by the addition of 5 µl 2-vinylpyridine for 1 h at room temperature. The amount of GSSG in 50 or 40 µl of GSH lysate was measured according to the method of Tietze, which is based on the reduction of 5,5′-dithiobis-(2-nitrobenzoic) acid (DTNB) (150 µM) [15], [33].

### Glutathione reductase and glutathione peroxidise activity assays

Glutathione peroxidise and glutathione reductase activities were analysed by spectrophotometric assays as described before [15], [34].

### TBRS assay

TBRS were detected using colorimetric assay as indicated before [35].

### Annexin V and DAPI stainings

Cells undergoing apoptosis were detected using Annexin V staining. Vybrant® apoptosis assay kit (Invitrogen, Paisley, UK) containing Annexin V conjugated with Alexa Fluor 488 was used for this analysis according to the manufacturer's protocol. 4′,6-diamidino-2-phenylindole diactate (DAPI, Invitrogen, Paisley, UK) staining was used according to the manufacturer's protocol to assess the total amount of cells. Fluorescent microscope (Nikon Eclipse 50i) was employed for visualisation and the images were captured using Image Pro Plus software.

### Statistical analysis

Each experiment was performed at least three times and statistical analysis was conducted using two-tailed Student's t test. Statistical probabilities (P) were expressed as *, where P<0.01.
